# Isolated Peroneal Nerve Palsy Following Bariatric Surgery in the Absence of Nutritional Deficiency: A Case Report

**DOI:** 10.7759/cureus.89459

**Published:** 2025-08-06

**Authors:** Rasha Ebrahim, Ijaz Kamal, Dania Akasha, Nidal Ahmed, Iyman Elsmani, Vamanjore A Naushad

**Affiliations:** 1 Department of Internal Medicine, Hamad Medical Corporation, Doha, QAT; 2 Clinical Department, College of Medicine-QU Health, Qatar University, Doha, QAT; 3 Department of Clinical Medicine, Weill Cornell Medicine-Qatar, Doha, QAT

**Keywords:** bariatric surgery, foot drop, laparoscopic gastric sleeve gastrectomy, peripheral neuropathy, unilateral peroneal nerve palsy

## Abstract

Peroneal neuropathy is a recognized cause for foot drop, typically following trauma, nerve damage, immobilization, or prolonged external pressure. Recently, rapid weight loss after bariatric surgery has been recognised as a potential cause for peroneal neuropathy. This may be due to the loss of protective fat tissue near the peroneal nerve, increasing its susceptibility to compression. Although this complication may be infrequent in occurrence, its impact on mobility and quality of life calls for increased vigilance among clinicians. Here we report a case of unilateral peroneal neuropathy leading to foot drop after significant weight loss in a short period following bariatric surgery. The patient underwent a comprehensive clinical workup, including laboratory investigations and neuroimaging, all of which were normal. Diagnosis was confirmed by nerve conduction studies, which revealed left peroneal nerve entrapment at the fibular head. Management consisted of a multidisciplinary, conservative approach, including physiotherapy and orthotic support.

## Introduction

Peroneal neuropathy is marked by weakness in dorsiflexion of the foot (foot drop) associated with sensory impairments along the lateral calf and dorsum of the foot. It can result from various causes, which include compression or trauma to the nerve, iatrogenic injury, and systemic disorders such as diabetes mellitus, malignancies, connective tissue disorders, or autoimmune diseases [1-4]. The common peroneal nerve, a branch of the sciatic nerve, is particularly susceptible to compression or entrapment as it wraps around the fibular head [4]. Certain medical interventions, such as bariatric surgery, can increase the risk of such nerve compression. In recent years, bariatric surgery has become increasingly common as a treatment modality for obesity. In the United States, over 260,000 procedures are performed annually, a 6.5% increase from 2021 to 2022 alone [5]. While the surgery offers substantial health benefits, including weight loss and improvement in obesity related complications, it also has its disadvantages. These include nutritional deficiencies, gastrointestinal issues, and peripheral neuropathies [6]. Rapid weight loss following bariatric surgery can lead to anatomical changes that make patients more vulnerable to nerve compression or entrapment, especially in regions where nerves pass through tight anatomical spaces [7]. The incidence rate of peripheral neuropathy following bariatric surgery is reported to be up to 16% [7]. Although rare, peroneal neuropathy has been identified as a possible complication of bariatric procedures, likely resulting from a combination of mechanical factors and metabolic alterations [8]. This case report presents a rare occurrence of unilateral peroneal entrapment neuropathy in a patient with rapid weight loss following bariatric surgery, emphasizing the importance of recognizing peripheral nerve complications in this patient group.

## Case presentation

A 49-year-old male smoker was admitted with a three-day history of left leg pain, numbness, and gait disturbances. He noticed an unusual sensation in his left foot for about seven days, which made it difficult to wear shoes comfortably, although there were no significant issues with the limb at that time. Three days before presentation, the patient developed progressive weakness in his left lower limb, more pronounced distally than proximally, along with numbness leading to multiple falls, prompting him to seek evaluation at the emergency department. There were no motor or sensory symptoms in other limbs. He denied back or neck pain, bowel or bladder incontinence, fever, headache, vomiting, or change in level of consciousness. There were no associated symptoms pertaining to other systems. 

Past medical history includes taking treatment for hypertension, hyperlipidemia, hypothyroidism, and coronary artery disease. He underwent gastric sleeve surgery seven months prior to presentation, and his preoperative body weight was 121.5 kg. After the surgery, he lost approximately 62 kg within the first three months. Postoperatively, he was prescribed multivitamin supplements and omega-3 ethyl ester capsules, to which he was compliant.

Physical examination of the left lower limb did not reveal tremor, fasciculation, myoclonus, or muscle wasting. Muscle tone was normal, and there was no ankle clonus. There was weakness of dorsiflexion (grade 1/5) and plantarflexion (grade 3/5) of the ankle joint. Deep tendon reflexes revealed a diminished Achilles reflex on the left, while other reflexes were normal. Sensory examination showed decreased pain and touch sensation below the left knee, more pronounced on the lateral aspect of the leg, consistent with L5 dermatome involvement. Neurological examination of other limbs did not reveal any positive signs. Babinski sign was negative bilaterally. The spine examination was normal. Gait assessment revealed a high-stepping gait, and when standing, the patient exhibited flexion of the left hip and knee, suggesting an anti-gravity posture. The left foot was in a foot drop position, and the Romberg test was negative. Examination of the other systems was unremarkable.

Routine blood tests were normal. The results of vitamin levels, minerals, iron studies, and autoimmune studies were all normal and did not reveal any abnormalities (Table 1). CT scan and MRI diffusion study of the head and MRI of the lumbar spine and sacrum were normal.

**Table 1:** Results of laboratory testing ANCA: anti-neutrophil cytoplasmic antibody; ANA: antinuclear antibody; CTD: connective tissue disease

Parameter	Values w/Units	Normal range
White blood count	6.9 x10^3/uL	4.0-10.0
Red blood count	5.7 x10^6/uL	4.5-5.5
Hemoglobin	16.8 gm/dL	13.0-17.0
Platelets	305 x(10^3)/uL	150-410
ESR	5 mm/hr	2-28
Urea	2.3 mmol/L	2.5-7.8
Creatinine	52 umol/L	62-106
Sodium	141 mmol/L	133-146
Potassium	4.1 mmol/L	3.5-5.3
Chloride	104 mmol/L	95-108
Bicarbonate	28 mmol/L	22-29
Bilirubin total	13 umol/L	0-21
Total protein	65 gm/L	60-80
Albumin level	36 gm/L	35-50
Alkaline phosphatase	87 U/L	40-129
Alanine transaminase	20 U/L	0-41
Aspartate transaminase	22 U/L	0-40
Iron	24 umol/L	6-35
Total iron binding capacity	45 umol/L	45-80
Transferrin	1.8 gm/L	2.0-3.6
Fe% saturation	53 %	15-45
Ferritin	209.0 ug/L	30.0-553.0
Copper level	17.9 umol/L	11.8-22.8
Zinc level	11.1 umol/L	10.1-16.8
Vitamin E	32 umol/L	12-42
Vitamin A	2.0 umol/L	1.0-2.1
Vitamin B1	160 nmol/L	66-201
Vitamin B6	38 nmol/L	20-121
Vitamin B12	253.0 pmol/L	145.0-569.0
Vitamin D	19 ng/mL	
Selenium	1.04 umol/L	0.89-1.90
Folate	17 nmol/L	10-70
Parathyroid hormone	34 pg/mL	15-65
Thyroid-stimulating hormone	3.86 mIU/L	0.30-4.20
Thyroxine	13.5 pmol/L	11.0-23.3
HbA1C %	5.8 %	
ANCA	Negative	
ANA CTD Int	Negative	
C3	1.05 gm/L	0.90-1.80
C4	0.26 gm/L	0.10-0.40

Nerve conduction velocity (NCV) evaluation of the left peroneal motor nerve showed decreased conduction velocity (Poplt-B Fib, 23 m/s). The left peroneal (TA) motor nerve showed prolonged distal onset latency (Poplit, 8.6 ms) and decreased conduction velocity (Poplit-Fib Head, 17 m/s). All the remaining nerve studies were within normal limits. Left vs. right side comparison data for the peroneal motor nerve indicated an abnormal left-right velocity difference (Poplt-B Fib, 37 m/s). All the remaining left vs. right side differences were within normal limits. F Wave studies indicated that the left peroneal F wave had no response, and the rest of the F Wave latencies were within normal limits. All F Wave left vs. right side latency differences were within normal limits. The nerve conduction study concluded that this was an abnormal study as there was electrophysiological evidence of left peroneal entrapment neuropathy (neuropraxic in nature) across the fibular head.

Bariatric medicine and neurology teams were consulted, and an empirical diagnosis of isolated peroneal nerve palsy was made due to significant weight loss post-bariatric surgery. The patient was started on empirical high-dose parenteral vitamins and minerals and received physiotherapy. After discharge, the patient followed up with a bariatric dietitian, physiotherapist, and the pedorthics team.

## Discussion

Bariatric surgery, specifically gastric sleeve surgery, has emerged as a highly effective treatment for obesity and metabolic syndrome worldwide. With the rising number of bariatric procedures performed, it is important to be aware of the early and late complications of this intervention. The overall incidence of complications is reported to be between 2-6.9% [9]. Early complications (<90 days) include anastomotic/staple line leaks, peritonitis, haemorrhage, infections, and DVT [10, 11]. Late complications (>90 days) include anastomotic stricture, bowel obstruction, nutritional deficiencies, and neurological complications [11]. Among these chronic complications, micronutrient deficiencies are common and require lifelong monitoring and supplementation. The incidence and type of nutritional deficiencies vary depending on the type of surgical procedure, with Roux-en-Y gastric bypass patients often experiencing deficiencies in calcium, iron, vitamin D, and B12 [12]. The occurrence of these deficiencies is also influenced by the patient’s adherence to nutritional supplementation and follow-up care. Overall, the safety profile of bariatric surgery has improved significantly with advancements in surgical techniques and postoperative care, but it remains crucial to perform these procedures in high-volume centers with an experienced surgical team to minimize risks.

Peroneal nerve palsy following bariatric surgery is a recognized but uncommon complication, typically presenting as a unilateral foot drop. Bilateral cases are rare but have been documented [13]. The incidence rate varies across studies. Approximately 0.5% of patients are reported to develop this condition following surgery [6]. However, a single-centre report from Spain, which included 1500 cases, found an even lower incidence of just 0.13%, highlighting the rarity of this complication [14]. The onset of symptoms generally occurs several months after the surgery, but can vary widely. In one published case report, a patient developed left-sided foot drop 18 weeks post-surgery, followed by right-sided involvement 24 weeks after the surgery [8]. Another study noted that symptoms appeared 5 to 11 months postoperatively in a total of 14 patients among 2607 patients who were retrospectively evaluated [6]. In the present case, the patient developed symptoms seven months after surgery. This variability in time duration between the surgery and the onset of symptoms could be multifactorial. These factors may include preoperative baseline characteristics of the patient, presence of comorbidities, medications, and postoperative status of the patient, including rapid weight loss or nutritional deficiencies.

Current literature regarding the pathophysiological mechanism for peroneal nerve palsy following bariatric surgery is multifactorial. However, the most common factor implicated is mechanical compression of the nerve. The common peroneal nerve passes superficially around the fibular head, protected by a layer of adipose tissue. Significant weight loss leads to a decrease in these protective fat pads and subcutaneous adipose tissue, leaving the nerve vulnerable to compression against bone or external factors [7,13]. Furthermore, the superficial location of the nerve with minimal surrounding protective muscles or soft tissues makes it anatomically vulnerable to external compression [13]. External contributors to peroneal nerve impingement may include habitual leg crossing and prolonged squatting [4,14]. Therefore, patients should be advised to avoid these positions to reduce the risk of nerve compression [6]. Other theories include loss of perineural and intraneural fat, microcirculatory nerve dysfunction with ischemia with or without oedema, metabolic and hormonal change that occurs following bariatric surgery increase the susceptibility of the nerve to compression and mechanical stress, resulting in inflammation, oedema, and ischemia, further leading to decreased nerve conduction and paralysis [15]. Furthermore, the relationship between BMI and fibular tunnel size, with the thickness of the nerve, is also implicated as a cause for the nerve damage [16]. In addition to the above causes, nutritional deficiencies may contribute to neuropathic symptoms. The suggested micronutrients include vitamins (B1, B12, E, D) and minerals (copper) [6-7]. However, these deficiencies more commonly cause polyneuropathy, and the unilateral peroneal nerve is more often linked to local mechanical compression [7,14].

Several case reports illustrate the multifactorial aetiology of peroneal neuropathy following rapid weight loss. Oh et al. described a case of bilateral peroneal neuropathy following weight loss (10 kg in 24 days) due to poor oral intake after biliary surgery, demonstrating that significant weight loss, regardless of the surgical type, may predispose patients to peripheral nerve compression [13]. Margulis et al. reported a case of bilateral common peroneal nerve entrapment in a patient who experienced significant weight loss over a short period (40 kg in 16 weeks) with normal laboratory workup, highlighting the fact that mechanical factors alone can cause neuropathy [17]. In contrast, a more recent case report by Lee et al. reported unilateral peroneal neuropathy after laparoscopic sleeve gastrectomy in a middle-aged woman with recurrent vomiting and pancreatic insufficiency, who was noncompliant with supplements, suggesting combined roles for rapid weight loss, nutritional deficits, and local nerve compression [18]. In the current case, the patient demonstrated adequate compliance with his nutritional supplements, and laboratory investigations did not reveal any micronutrient deficiency. Considering the rapid amount of weight (62 kg) loss within a short period, mechanical compression is the most likely reason for the nerve palsy. This aligns with the previously reported cases that demonstrated rapid weight loss alone, even in the absence of nutritional deficiency, can cause peroneal nerve palsy in bariatric surgery patients.

The extent and rapidity of weight loss have also been implicated as a risk factor for peroneal neuropathy. Weyns et al. reported that peroneal nerve palsy can occur when body fat decreases by > 10%, and Shahar et al. showed that rapid weight loss is associated with peroneal neuropathy [19,20]. Meylaerts et al. compared patients who developed foot drop following bariatric surgery with a control group who did not develop foot drop, and reported that the mean weight loss in patients with peripheral neuropathy was 45 kg over a mean period of 8.6 months whereas, in the control group the mean weight loss was 43.8 kg over a mean period of 21.7 months [16]. This signifies the importance of the time period of weight loss as a risk factor for peroneal neuropathy.

The management of peroneal nerve palsy includes a multidisciplinary approach with lifestyle modifications, nutritional support, weight management, and physical therapy, which are often effective [2]. These measures are crucial for improving the patient’s muscle strength and mobility, as well as preventing further complications. In some cases, where symptoms persist, more invasive interventions like surgical decompression may be necessary [14]. The importance of maintaining adequate nutritional support post-bariatric surgery cannot be overstated. While some deficiencies can contribute to neuropathy, compliance with supplementation and nutritional monitoring can significantly reduce the risk of complications. This aligns with the findings of Lee et al. and Ramos-Leví et al., which emphasize the need for an individualized care plan that includes attention to both physical and psychological well-being [14,18]. Unilateral peroneal nerve palsy usually has a high recovery rate. Most cases resolve completely, particularly if the nutritional status is optimized and further rapid weight loss is reduced [8,14]. In terms of management, the approach in the present case was conservative, including physical therapy, orthotic devices (such as ankle-foot orthoses), and careful monitoring of nutritional status.

## Conclusions

This case highlights the significance of recognising peripheral neuropathy as a potential complication of bariatric surgery, particularly in patients who experience rapid weight loss in a short period of time. Such Patients should be monitored for neuropathic symptoms, or if in doubt early electromyogram or NCV studies should be considered, as early diagnosis is crucial for a favourable neurological outcome and preventing long-term disability. Awareness and education among patients regarding gradual weight loss might be of help.
